# Cancer-induced bone pain sequentially activates the ERK/MAPK pathway in different cell types in the rat spinal cord

**DOI:** 10.1186/1744-8069-7-48

**Published:** 2011-07-01

**Authors:** Li-na Wang, Ming Yao, Jian-ping Yang, Jun Peng, Yan Peng, Cai-fang Li, Yan-bing Zhang, Fu-hai Ji, Hao Cheng, Qi-nian Xu, Xiu-yun Wang, Jian-ling Zuo

**Affiliations:** 1 Department of Anesthesiology, The First Affiliated Hospital of Soochow University, Suzhou, 215006, China; 2 Department of Anesthesiology, The First Affiliated Hospital of Jiaxing University, Jiaxing, 314000, China; 3 The Library of the Second Military Medical University, ShangHai, 200433, China; 4 Brain research laboratory, The First Affiliated Hospital of Soochow University, Jiangsu 215006, China

**Keywords:** bone cancer pain, hyperalgesia, spinal cord, extracellular signal-regulated protein kinase (ERK), cAMP response element-binding protein (CREB), rat

## Abstract

**Background:**

Previous studies have demonstrates that, after nerve injury, extracellular signal-regulated protein kinase (ERK) activation in the spinal cord-initially in neurons, then microglia, and finally astrocytes. In addition, phosphorylation of ERK (p-ERK) contributes to nociceptive responses following inflammation and/or nerve injury. However, the role of spinal cells and the ERK/MAPK pathway in cancer-induced bone pain (CIBP) remains poorly understood. The present study analyzed activation of spinal cells and the ERK/MAPK pathway in a rat model of bone cancer pain.

**Results:**

A Sprague Dawley rat model of bone cancer pain was established and the model was evaluated by a series of tests. Moreover, fluorocitrate (reversible glial metabolic inhibitor) and U0126 (a MEK inhibitor) was administered intrathecally. Western blots and double immunofluorescence were used to detect the expression and location of phosphorylation of ERK (p-ERK). Our studies on pain behavior show that the time between day 6 and day 18 is a reasonable period ("time window" as the remaining stages) to investigate bone cancer pain mechanisms and to research analgesic drugs. Double-labeling immunofluorescence revealed that p-ERK was sequentially expressed in neurons, microglia, and astrocytes in the L4-5 superficial spinal cord following inoculation of Walker 256 cells. Phosphorylation of ERK (p-ERK) and the transcription factor cAMP response element-binding protein (p-CREB) increased in the spinal cord of CIBP rats, which was attenuated by intrathecal injection of fluorocitrate or U0126.

**Conclusions:**

The ERK inhibitors could have a useful role in CIBP management, because the same target is expressed in various cells at different times.

## Background

Prevention and control of cancer-induced bone pain (CIBP) is one of the most difficult tasks for pain management practitioners, although pain is very common in bone cancer patients [1]. Currently, pharmacological treatments for chronic pain are based on the understanding of mechanisms of drug action in non-cancer pain syndromes. However, the treatments do not target specific neurobiological changes in CIBP. To properly evaluate the current therapies and development of novel therapies, it is important to understand the underlying mechanisms of CIBP.

Glial cells are classically viewed as central nervous system (CNS) cells that passively provide a variety of important metabolic and structural roles to support neurons and do not actively participate in information processing. However, recent studies have demonstrated the critical importance of glial cells in a variety of biological functions, including pain perception and modulation [2-5]. Astrocytes and microglia in the spinal cord participate in initiation and maintenance of persistent pain induced by tissue inflammation and nerve injury. Peripheral nerve injury induces neuropathic pain and phosphorylation of mitogen-activated protein kinase (MAPK) family members in dorsal root ganglia (DRG) and the dorsal horn. Following nerve injury, phosphorylation of extracellular signal-regulated protein kinase (ERK), an important member of the MAPK family, increases sequentially in neurons, microglia, and astrocytes of the dorsal horn. Nerve injury-induced phosphorylation of ERK (p-ERK) occurs early and is long-lasting, and in several animal models of neuropathic pain, MEK inhibitors, which are known to suppress ERK activation, have proven effective in pain alleviation at various time points [6-8]. Spinal nerve ligation (SNL) induces a particular temporal pattern of ERK activation in the spinal cord-initially in neurons, then microglia, and finally astrocytes [9]. ERK likely contributes to neuropathic pain through various mechanisms in different cell types at different times. Therefore, ERK/MAPK regulation is a promising therapeutic target for treatment of neuropathic pain. However, the role of spinal cells and the ERK/MAPK pathway in bone cancer pain remains poorly understood, although CIBP is a unique state with features of neuropathy and inflammation.

In a previous study from our laboratory, a rat model of bone cancer pain [10-12] was established using female Sprague Dawley (SD) rat carcinoma Walker 256 cells according to a previously described method [13-17]. This animal model was further utilized to demonstrate activation of three types of spinal cells and the ERK/MAPK pathway in the spinal cord of CIBP rats and to assess the role of the ERK/MAPK pathway in chronic bone cancer pain.

## Results

### Radiological and histochemical analysis of tumor development in the tibia

**Figure 1:**
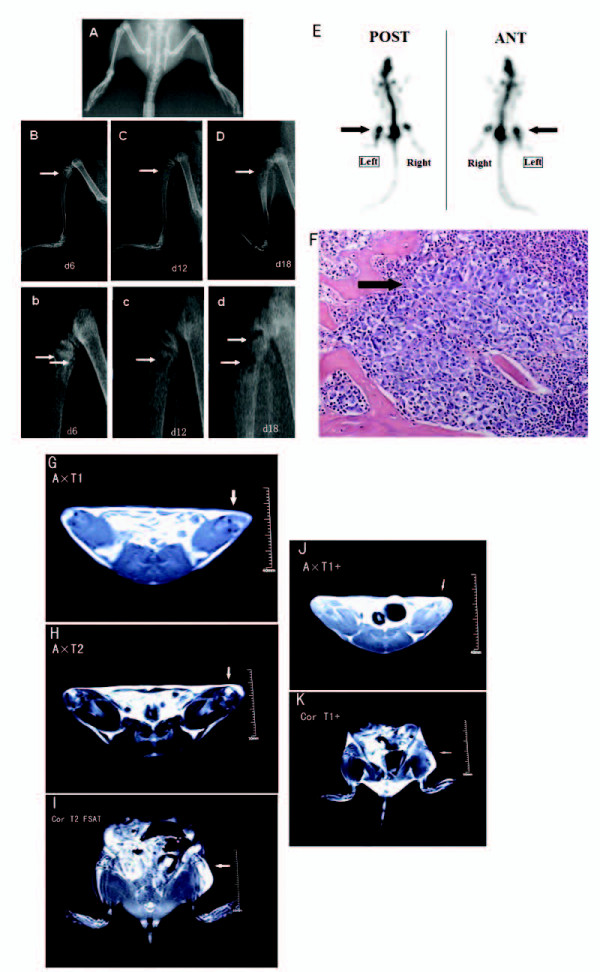
**Radiological and histochemical analysis of tumor development in the tibia**. **(A-D)** To confirm bone destruction of the tibia by tumor, rats were radiographed on 6 (Figure B and b), 12 (Figure C and c) and 18 days (Figure D and d) after carcinoma cell inoculation of the ipsilateral hind limb. Arrows indicate structural destruction of the proximal cortical bone. In addition, for the control, bilateral hind limb of group K1 rats on days 18 was showed in Figure A. **(E) **Whole body bone SPECT imaging at day 12 post-injection was showed in Figure E. SPECT detected bone formation to be active in the left proximal tibia (see the arrows). **(F) **HE staining of the left tibia showed that bone marrow spaces were infiltrated with malignant tumor (Cancer nest, see the arrow) on day 12 after Walker 256 cell inoculation. **(G-K) **On day 18 after carcinoma cell inoculation, rats were also investigated with MRI (Magnetic Resonance Imaging) of the hind limbs (Figure G-K). After optimal adjustment of contrast, axial T1-weighted imaging (A × T1, Figure G), axial T2-weighted imaging (A × T2, Figure H), coronal fat suppressed sequence T2-weighted imaging (Cor × T2+, Figure I), axial enhanced T1-weighted imaging (A × T1+, Figure J), and coronal enhanced T1-weighted imaging (Cor × T1+, Figure K) data were analyzed by visually identifying and encircling areas of abnormal signal intensity for each MR section using a side to side comparison on the screen. Full thickness bicortical bone loss, cortical destruction, and soft tissue tumors were observed obviously (Arrow).

Bone destruction was monitored using radiological (SPECT, X-ray and MRI) and histological methods (Figure 1). Radiological analysis revealed decreased left hind limb activity and minute bone trabecula defects in the proximal epiphysis at 6 days after inoculation in group V1 (*in vitro *Walker 256 cells) and group A1 (*in vivo *Walker 256 cells) rats (Figure 1-B, b). Further deterioration was detected at 12 days post-injection, with full thickness unicortical bone loss, according to radiological analysis (Figure 1-C, c), as well as bone formation in the left proximal tibia, as detected by SPECT (Figure 1-E). SPECT analysis provided better contrast and more accurate detection and localization of lesions compared with planar scintigraphy, which demonstrated that SPECT scanning has value in the diagnosis of bone metastatic cancer [18]. Rats from group A1 and group V1 exhibited weight loss by day 15(data not shown). In addition, on day 18 after cell injection, full-thickness, bicortical, bone loss, as well as cortical destruction and soft tissue tumors [not detected by X-ray (Figure 1-DV), but by MRI], was observed by X-ray (Figure 1-D, d) and MRI (Figure 1-G-K). At this time, because of the occurrence of soft tissue tumors, it was assumed that extra-osseal tumor growth was limited to the proximal epiphysis. However, in the following days, further bone erosion and extra-osseal tumor dissemination were observed on day 21 following inoculation (data not shown). Therefore, it is presumed that day 6-18 post-inoculation is a reasonable time window for evaluating anti-nociceptive agents in this animal model. In addition, progressive local bone destruction takes place at the proximal epiphysis of the tibia during this time.

Histological examination revealed bone marrow spaces infiltrated with malignant tumor on day 12 after inoculation (Figure 1-F). Bone destruction was not observed in the vehicle or sham group animals.

### Mechanical allodynia

**Figure 2:**
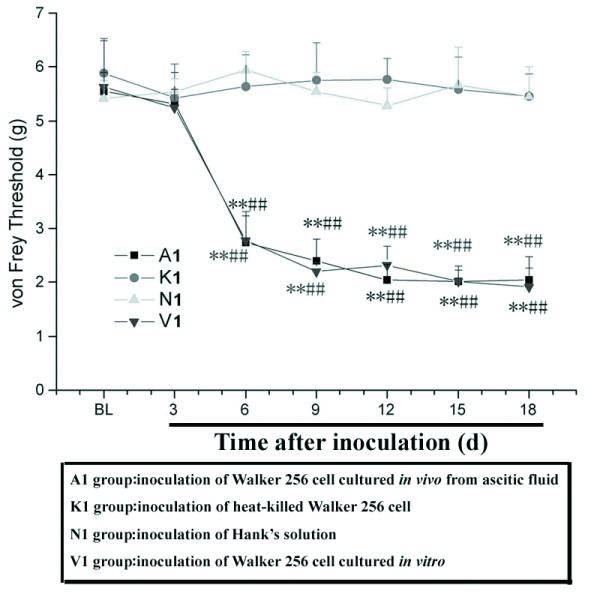
**Mechanical allodynia (von Frey filaments)**. The 50% paw withdrawal threshold (PWT) to a static mechanical stimulus was assessed using von Frey filaments and the up-and-down method. Rats received intra-tibial inoculations of two kinds of Walker 256 cell suspension (1 × 10^5 ^cells/5 μl Hank's solution) (group V1 and group A1), heat-killed carcinoma cells in 5 μl Hank's solution (group K1), or 5 μl Hank's solution only (group N1). PWT was measured pre-surgery (Baseline, BL) and on days 3, 6, 9, 12, 15, and 18 after cell inoculation (Figure 2). All data are expressed as means ± SEM. **P < 0.01 *vs*. group N1. ## *P *< 0.01 *vs*. group K1.

Following Walker 256 cell inoculation of the tibia (groups V1 and A1), median paw withdrawal threshold (PWT) of the inoculated hind paw progressively decreased, and the contralateral hind paw remained unchanged (data not shown). The overall difference between the two hind paws was significant (*P *< 0.05, data not shown). Dunn's multiple comparisons test revealed that median PWT from the inoculated paw was significantly less than the contralateral paw or the control group between day 6 and 18 post-cell inoculation (*P *< 0.01, Figure 2). These results suggested development of mechanical allodynia in the inoculated hind paw.

In this studies, Walker 256 cells, cultured *in vitro *and *in vivo*, were utilized, and above methods and results demonstrated that cells cultured *in vivo *and isolated from ascitic fluid may be more user-friendly. Moreover, at the beginning of this period (day 6), mechanical hyperalgesia and allodynia were evident, which was earlier or later than previously reported [day 10 to 14 [13], and day 4 [14]], respectively. This difference was likely due to differences in invasiveness and rat sexes of MRMT-1 cells compared with Walker 256 cells.

### Anti-nociceptive effect of intrathecal FC and U0126

**Figure 3:**
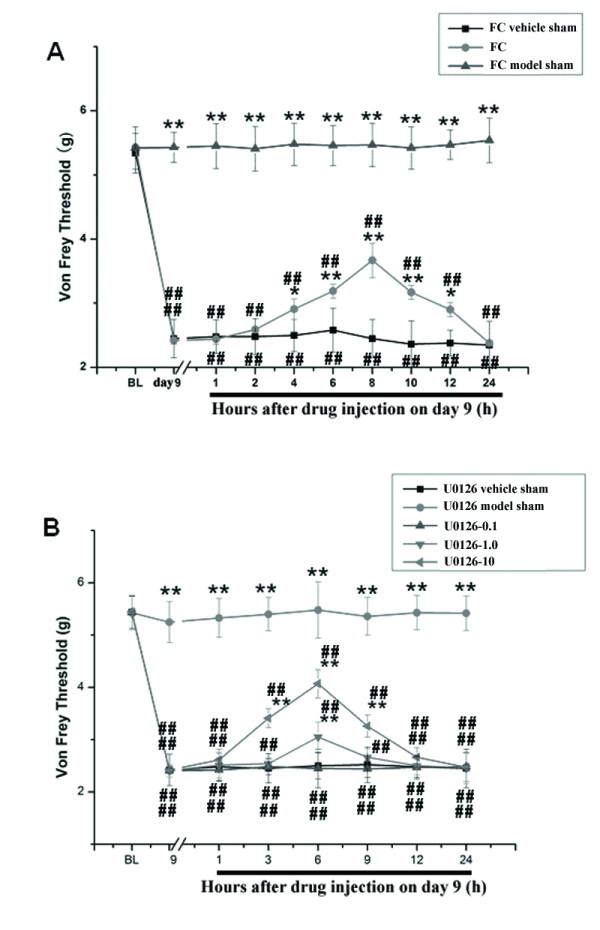
**Anti-nociceptive effect of intrathecal FC and U0126**. **(A) **The von Frey threshold in group FC was significantly increased 4-12 h post FC intrathecal injection compared with that in group FC vehicle sham (Figure 3A). All data are expressed as means ± SEM. *P < 0.05, ** *P *< 0.01 *vs*. group FC vehicle sham. ## *P *< 0.01 vs. group FC model sham. **(B) **Three doses of U0126 (0.1, 1.0 and 10 μg) were compared (Figure 3B). 1 μg (group U0126-1.0)and 10 μg (group U0126-10) U0126 significantly attenuated the mechanical allodynia induced by bone cancer pain, but 0.1 μg (group U0126-0.1) U0126 had no effect. 1 μg U0126 (group U0126-1.0) attenuated the mechanical allodynia for 6 h after administration, while 10 μg U0126 (group U0126-10) attenuated the mechanical allodynia for 9 h after treatment. This suggested that intrathecal injection of 10 μg U0126 may relieve pain, for up to 9 h after administration. *P < 0.05, ** *P *< 0.01 *vs*. group U0126 vehicle sham. ## *P *< 0.01 *vs*. group U0126 model sham.

The von Frey threshold in group FC significantly increased at 4-12 h post-FC intrathecal injection compared to groups FC vehicle sham and FC model sham (*P *< 0.01, Figure 3A), with a peak anti-nociceptive effect of 8 h. GFAP-positive astrocytes and OX-42-microglia were atrophied and were more ramified in group FC compared with groups FC vehicle sham and FC model sham (*P *< 0.01, data not shown).

Three doses of U0126 (0.1, 1.0, and 10 μg) were compared, revealing that 1 μg (group U0126-1.0) and 10 μg (group U0126-10) U0126 significantly attenuated CIBP-induced mechanical allodynia. However, 0.1 μg (group U0126-0.1) U0126 had no effect. In addition, 1 and 10 μg intrathecal U0126 significantly reduced PWT compared with the control group (*P *< 0.05, Figure 3B). Although 1 μg U0126 attenuated mechanical allodynia for 6 h after administration, 10 μg U0126 attenuated mechanical allodynia for 9 h, which demonstrated that intrathecal injection of 10 μg U0126 could relieve pain for up to 9 h after administration.

### CIBP-induced sustained activation of p-ERK and p-CREB in the spinal cord

**Figure 4:**
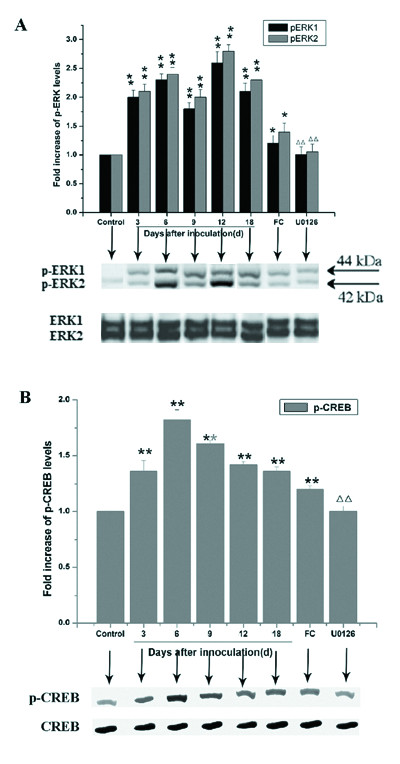
**Sustained activation of p-ERK and p-CREB in the spinal cord induced by CIBP**. The p-ERK (Figure 4A) and p-CREB (Figure 4B) were tested through Western blots on day 3, 6, 9, 12, 18 (n = 5 for each time group) after cell inoculation. This analysis revealed an activation of p-ERK and p-CREB in the L4-5 spinal dorsal horn from day 3 although at a low level, and that significantly increased (*P *< 0.05) at other time points (day 6, 9, 12, 18) examined after CIBP. *P < 0.05, ** *P *< 0.01 *vs*. control (group N1), group FC model sham, and group U0126 model sham. ^Δ^*P *< 0.05, ^ΔΔ^*P *< 0.01 *vs*. group U0126 vehicle sham, and group U0126 model sham.

p-ERK (Figure 4A) and p-CREB (Figure 4B) were assessed by Western blot analysis on day 3, 6, 9, 12, and 18 (n = 5/time group) after cell inoculation. Analysis revealed low levels of p-ERK and p-CREB activation in the L4-5 spinal dorsal horn at day 3, and that significantly increased (*P *< 0.05) activation at other time points (day 6, 9, 12, and 18) following CIBP.

At day 9, rats were sacrificed 6 h after U0126 (10 μg) or vehicle (5% DMSO) intrathecal injection, or at 8 h after FC or vehicle (0.3% 2 M HCl/PBS) injection. The time points (6 and 8 h) were based on or pain-related behavioral results of peak anti-nociceptive effects. Injection of U0126 or FC induced a rapid decrease in p-ERK and p-CREB activation. From this time point on, a slow, irregular decline of p-ERK and p-CREB expression occurred (data not shown).

These results demonstrated decreased spinal ERK and CREB activation following intrathecal administration of U0126 or FC, which was consistent with previous anti-nociceptive paradigms.

### Sequential p-ERK activation in various cell types

**Figure 5:**
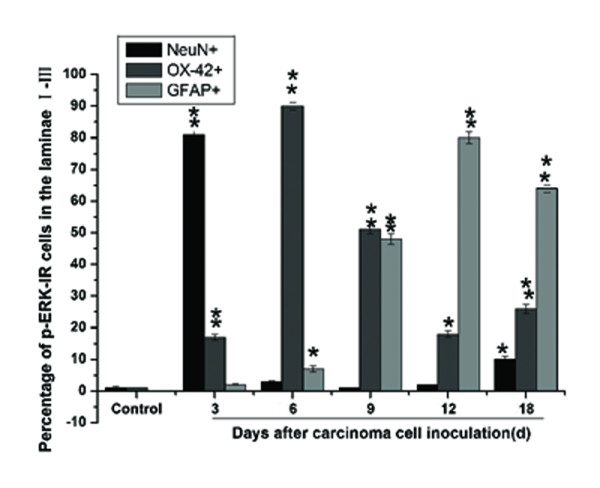
**Percentage of double-labeling of p-ERK-IR cells**. The percentage of double-labeling of p-ERK-IR with NeuN, OX-42, or GFAP-IR in the spinal dorsal horn (laminae I-III) on different days (from day 3 to day 18) after carcinoma cells inoculation. About 200-250 p-ERK-IR cells were counted in each condition. The percentage of double-labeling = the number of double-labeled cells/number of p-ERK-labeled cells. *P < 0.05, ** *P *< 0.01 *vs*. control (group N1).

**Figure 6:**
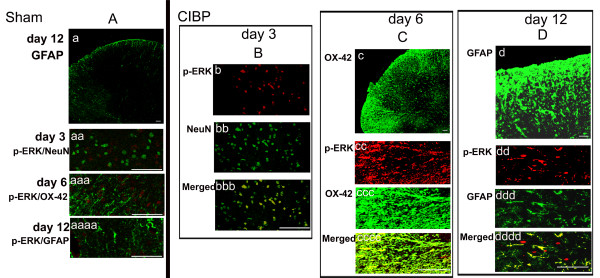
**Sequent activation of p-ERK in different cell types respectively**. On day 3 after carcinoma cell inoculation, moderate p-ERK immunoreactive (IR) neurons (Figure 6B) were found in ipsilateral spinal cord which were much more than that of the control (Figure 6A-aa). The p-ERK is colocalized with NeuN (Figure 6B-bb, green), and the double-labeling cells (Figure 6 B-bbb, yellow) were mainly located in the superificial dorsal horn (laminae I-II) of the ipsilateral L4-5 spinal cord. The relative peak of p-ERK-IR microglia was on the day 6 after carcinoma cell inoculation. The microglia p-ERK activation (Figure 6C-cccc, yellow) was widespread in the dorsal and ventral horns. Almost all p-ERK-IR cells (Figure 6C-cc, red) in the L4-5 spinal cord expressed OX-42 (Figure 6C-ccc, green) which were much more than that of the control (Figure 6A-aaa). Moreover, on day 12, double staining indicated that the p-ERK (Figure 6D-dd, red) was predominantly localized in GFAP-IR astrocytes (Figure 6D-ddd, green).

At day 3 after carcinoma cell inoculation, p-ERK immunoreactive (IR) neurons were detected in the ipsilateral spinal cord (Figure 6B-b, red), and the number of p-ERK-positive cells was greater than in the contralateral (data not shown) or control groups (Figure 6A-aa) (including group with heat-killed cells, Group K2). The p-ERK/NeuN double-labeled cells were located in the superficial dorsal horn (laminae I-II) of the ipsilateral L4-5 spinal cord. Neuronal p-ERK activation decreased to a lower level (1-3 cells/section) in the deep (lamina III-X) dorsal horn compared to controls on day 12. Interestingly, On day 18 the neuronal p-ERK was little activated again in the medial dorsal horn (laminae I-III) although that was lower than the levels on day 3 (Figure 5). This could be due to late dorsal horn p-ERK activation, as discussed in the following.

Co-expression of p-ERK/OX-42 also occurred on day 3 and day 18 (Figure 5) following carcinoma cell inoculation, although expression was less than p-ERK/NeuN on day 3 and greater than the contralateral side (data not shown) and in the control group (Figure 6A-aaa) (including Group K2). These results were not consistent with previous results [9], which demonstrated no co-expression of p-ERK with either OX-42 or GFAP in the early stage of model establishment (10 min for SNL model). Further, results from the present study revealed a relative peak for p-ERK-IR microglia at day 6 (Figure 5 and 6) after carcinoma cell inoculation. In addition, there was a substantial increase p-ERK expression in the ipsilateral L4-5 spinal cord from day 3 to 9. This sequential microglia p-ERK activation was widespread in the dorsal and ventral horns. The majority of p-ERK-IR cells in the L4-5 spinal cord expressed OX-42 (Figure 6C) at day 6 post-inoculation, with very few p-ERK-IR cells expressing NeuN and GFAP. In addition, on day 9, p-ERK was rarely detected in neurons, but remained moderately present in microglia of the deep dorsal horn (laminae III-V).

From day 9 onwards, co-expression of p-ERK/GFAP slowly increased. GFAP-positive p-ERK expression was detected primarily in the superficial dorsal (laminae I-II) of the ipsilateral L4-5 spinal cord. On day 12, double-labeling revealed predominant p-ERK expression in GFAP-IR astrocytes (Figure 6D). Moreover, p-ERK was expressed in some OX-42-IR cells in the deep dorsal horn, although there was very little p-ERK/OX-42 co-expression in the medial dorsal horn (laminae I-III).

## Discussion

The present study successfully established a female bone cancer pain model again, modified from that described by Medhurst *et al. *[13-15]. Studies on pain behavior, including mechanical allodynia (PWT) and spontaneous pain (ambulatory score), from our laboratory demonstrated that between day 6 and 18 is a reasonable period ("time window") to investigate bone cancer pain mechanisms and the effect of analgesic drugs. In addition, after injection of Walker 256 cells into the right tibia, left hind paw PWPT (nociceptive hind paw withdrawal pressure threshold) [data is presented in our previous study, [10]] also significantly and progressively decreased from days 6 to 18. But following injection of Walker 256 cells, the left hind paw withdrawal latency (PWL) following a thermal nociceptive stimulus only significantly decreased between days 3 and 6 compared with that of the contralateral hind paw and of the hind paws of control, which remained at the pre-injection level (see Additional file 1). However, *post-hoc *means comparisons revealed that Walker 256 cell inoculation of the tibia induced no significant (*P *> 0.05) decrease of PWL on days 9, 12, 15 and 18 after inoculation compared with groups N1 and K1. This is different from previous studies in CIBP [14]. But importantly, this is also observed in the animals with the heat killed cells, indicating a non-cancer effect at this time-point. We speculated that the immune system may play a role in which. The reason for this is unclear and is worthy of further study.

The MAPK family includes ERK, p38MAPK, and c-Jun N-terminal kinase (JNK) [7]. Initiation of the ERK/MAPK cascade involves activation of three kinases: Ras→Raf→MEK→ERK/MAPK, and the ERK/MAPK pathway is traditionally thought to play important roles in cell proliferation and differentiation [19-22]. Recently, ERK/MAPK activation was shown to contribute to nociceptive responses in the dorsal horn and DRG following inflammation and/or nerve injury [7,23]. Following nerve injury [9], p-ERK levels sequentially increase in neurons, microglia, and astrocytes of the dorsal horn. In addition, nerve injury-induced p-ERK occurs early and is long lasting. Moreover, previous studies [6,24,25] reported that U0126 prevents early increases in CREB phosphorylation in the superficial dorsal horn of chronic constriction injury (CCI) or arthritic rats, indicating that ERK/MAPK phosphorylation is likely an upstream signaling event that regulates CREB activation in these models [7]. In our present study, three doses of U0126 (0.1, 1.0, and 10 μg) were compared. Results demonstrated that intrathecal injection of 1.0 μg (group U0126-1.0) or 10 μg (group U0126-10) U0126 relieved pain for up to 9 h after administration. The p-ERK and p-CREB activation as detected in the L4-5 spinal dorsal horn starting at day 3, although at a low level, which significantly increased at other time points (day 6, 9, 12, and 18) after CIBP. This change was attenuated by an intrathecal injection of FC and U0126 on day 9 post-inoculation, which indicated that spinal ERK/MAPK pathway activation decreases with intrathecal administration of drugs in existing anti-nociceptive paradigms.

ERK activation plays an important role in induction and maintenance of neuropathic and inflammatory pain through different cellular mechanisms in the spinal cord [5]. A previous study [9] examined ERK activation pattern in the spinal cord at different time points (2, 10, and 21 days) after SNL, demonstrating sequential activation of ERK in the spinal cord after SNL. These results support a role for microglia in the initiation phase and a role for astrocytes in maintaining hypersensitivity. Different from SNL, CIBP is that the inflammation, tumor-released products, and tumor-induced injury to primary afferent neurons may simultaneously drive this chronic pain state. However, the mechanism responsible for the induction and maintenance of bone cancer pain is not clearly understood. What is in some ways unique about CIBP is that glial activation patterns in the spinal cord are quite different. Inflammation, tumor-released products, and tumor-induced injury of primary afferent neurons simultaneously drive the chronic pain state. CIBP is rarely only neuropathic, inflammatory, ischemic, or visceral, but is rather a combination of these. Clinically, CIBP [1] exhibits the hallmarks of cancer pain: the presence of disease with no pain at some sites and severe pain at others. It remains unclear as to why some areas of cancer growth and destruction induce pain, while others do not, and why some are painful with no apparent alteration in the level of damage or tumor load.

In this study, double-label immunofluorescence demonstrated that p-ERK was sequentially and respectively expressed in neurons, microglia, and astrocytes of the L4-L5 superficial spinal cord following inoculation of Walker 256 cells. These results were consistent to previously reported neuropathic pain models [9]. However, the microglia were also activated at day 3, with a substantial increase from day 3 to 9, which was different from previous CIBP results [5]. The reason for this discrepancy remains unclear and is worthy of further study. The difference of animals (rats *vs *mice) and carcinoma cell lines (Walker256 cells *vs *NCTC cells) maybe one of the reasons.

Pain is associated with altered gene expression in primary afferent neurons and second-order spinal cord neurons [5]. Results from the present study revealed moderate p-ERK expression in the ipsilateral spinal cord on day 3 after carcinoma cell inoculation. The vast majority of primary afferents, which make synaptic contacts with spinal dorsal horn neurons, contain glutamate, which is released by activity. Glutamate, substance P (SP), and calcitonin gene-related peptide (CGRP) exert excitatory effects, which lead to depolarization of spinal neurons. ERK activation may be an initial trigger of spinal neurons activation. In addition, reduced, but sustained, ERK activation is present in dorsal horn neurons for several weeks, which could play a role in maintaining gene expression.

Furthermore, microglial activation occurs following injury to peripheral nerves [9] or CIBP. The present results revealed a relative peak of p-ERK-expressing microglia on day 6 after carcinoma cell inoculation. In addition, there was a substantial increase in the ipsilateral L4-5 spinal cord from day 3 to 9. Microglial-mediated sensitization could be induced through release of nerve sensitizing factors, such as brain-derived neurotrophic factor (BDNF), interleukin (IL)-1β, tumor necrosis factor (TNF)- α, nitric oxide (NO), and prostaglandins [5]. A recent study from our laboratory [11] demonstrated a critical role in CNS innate immunity for the microglial Toll-like receptor 4 (TLR4) in induction and maintenance of behavioral hypersensitivity in a rat model of bone cancer pain. It is hypothesized that TLR4 could serve as the main mediator in induction of bone cancer pain. Further study of this early, specific, and innate CNS/microglial response, and how it leads to sustained glial/neuronal hypersensitivity, could lead to novel therapies for prevention and treatment of bone cancer pain syndromes.

Interestingly, on day 18, neuronal and microglia p-ERK were slightly activated in the medial dorsal horn (laminae I-III). It is possible that bone cortical destruction and soft tissue tumors could lead to increased primary afferent input (peripheral sensitization), as well as hyperactivity of spinal dorsal horn neurons (central sensitization).

Results demonstrated that p-ERK/GFAP co-expression increased slightly starting at day 9, but was significantly activated by day 12. Astrocytes are located very close to neurons and are sensitive to alterations in neuronal environments. Substances released by primary afferent neurons (glutamate, SP, CGRP, ATP, and NO) and microglia (BDNF, IL-1β, and TNF-α) can activate astrocytes, which supports a role for astrocytic p-ERK in the maintenance of late-phase CIBP.

Moreover, in this article, the discrepancy change of CREB-ERK1/2 phosphorylation and behavioural test was also found on day 3. The inconsistent findings may be due to the following reason: In this CIBP model, the PWT (mechanical allodynia) was also reduced on day 3, while there was no statistical difference between the normal control and cancer bone pain group. Importantly, this is also observed in the animals with the heat killed cells, indicating a non-cancer effect at this time-point. We speculated that the immune system may play a role in which. Moreover, radiographs showed no obvious bone destruction in the tibia, indicating that tumor cells did not metastasize at this time point. In addition, the neuronal mechanisms that turn pain signals into behavioral disorders are far from understood. It has been reported by Zhuang [9] et al. that acute spinal nerve ligation increases p-ERK level in the spinal dorsal horn, and this activation has been found within 10 min while at this time the mechanical allodynia should not occur. The reason for this discrepancy change is unclear and is worthy of further study.

## Conclusion

The ERK inhibitors could have a useful role in CIBP management, because the same target is expressed in various cells at different times [7,9]. In addition, inhibition of a single target alters the function of neurons, microglia, and astrocytes. Future studies should detail the pathophysiological events that activate the ERK/MAPK pathway and spinal cells to advance the understanding of the role of ERK phosphorylation and spinal cells in mechanisms underlying bone cancer pain.

## Methods

### Animals

Female SD rats, weighing 180-200 g, were maintained under controlled conditions (22-24°C, relative humidity 40-60%, 7:00 am-7:00 pm alternate light-dark cycles, and free access to food and water). All experiments were conducted in accordance with NIH guidelines and the regulations of the Soochow University Committee for the care and use of laboratory animals, and with the approval of local Ethics Review Board [26].

### Cell preparation

The Walker 256 mammary gland carcinoma cell line, which are syngenic with the SD rat, were cultured *in vitro *as previously described [10-12]. For injection, the cells were collected and diluted to a final concentration of 2 × 10^7 ^cells/ml and kept on ice until injected into rats.

For *in vivo *production, a total of 0.5 ml Walker 256 cells (2 × 10^7 ^cells/ml) were injected into the abdominal cavity of SD rats. After 7-14 days, the ascites (approximately 50-150 ml) were collected. The cells were collected by centrifugation for 3 min at 1200 rpm, and the pellet was washed three times with 10 ml D-Hank's solution and re-centrifuged for 3 min at 1200 rpm. Further, the cells were prepared as the above *in vitro*.

Two types of Walker 256 cell preparations, *in vitro *and *in vivo*, were injected into the tibial medullary cavity *via *the intercondylar eminence. 10 μl of cells were injected (approximately 1 × 10^5 ^cells/10 μl Hank's solution) into each rat. For the sham group, Walker 256 cells were prepared to the same final concentrations and boiled for 20 min.

### Surgical procedures

The surgery (n = 8/group) was committed as described previously [10-12]. All rats were anesthetized with sodium pentobarbital (45 mg/kg, i.p.). During the procedures, the Walker 256 cells (1 × 10^5 ^cells/10 μl Hank's solution) were then cultured *in vitro *(group V1), in ascitic fluid *in vivo *(group A1), heat-killed carcinoma cells in 10 μl Hank's solution (group K1), or 10 μl Hank's solution only (group N1) and were slowly injected into the cavity of the left tibia. Finally, all animals were allowed to recover from the inoculation surgery for 3 days prior to any experimentation.

Five days before intra-tibial injection of Walker256 cells, according to the modification of a method described previously [10-12], an intrathecal catheter (PE-10 tube) was inserted through the gap between the L3 and L4 vertebrae. The volume of dead space of the intrathecal catheter was 10 μl. To avoid occlusion of the catheter, 10 μl of normal saline was injected *via *a catheter on alternate days until the end of the experiment. The cannulated rats were allowed to recover for 3-4 days.

### Radiological bone examination

To determine tibial destruction from the inoculated tumor, rats were radiographed at 6, 12, and 18 days following carcinoma cell inoculation. Rats were placed on clear plexiglass and were exposed to an X-ray source under sodium pentobarbital anesthesia (45 mg/kg, i.p.). Using a Kodak Digital Radiographer System (Kodak, USA), tibial radiographs were taken (40 KVP, 2 ma) from hind limbs of groups V1, A1, K1, and N1 (n = 3/group).

In addition, at 12 days after carcinoma cell inoculation, single photo emission-computed tomography (SPECT) was used to determine alterations in local bone metabolism. The rats were anesthetized and placed in a prone position on the surgery table. A total of 0.2 ml ^99^Tc^m^-MDP (methylene diphosphonate, 5 mCi, or 185 MBq per injection) was injected into the tail vein, and dynamic data and images of hind limbs were collected, during blood flow perfusion phase and after 3 hours. Images and 5-minute dynamic data were collected during the bone phase.

Magnetic Resonance Imaging (MRI) was used to analyze hind limbs at 18 days after carcinoma cell inoculation (n = 3 rats/group). Following complete anesthesia induction with sodium pentobarbital (45 mg/kg, i.p.), gadolinium-DPTA [0.5 ml (235 mg), Gd-DTPA] was injected into the tail vein at 15 minutes after MRI. Following optimal adjustment of contrast, axial T1-weighted imaging (A × T1, TR:500.00 ms/TE: 12 ms/5 mm/Fov 12 cm/256 × 256/2), axial T2-weighted imaging (A × T2, TR:4221.00 ms/TE:106.2 ms/5 mm/Fov 12 cm/512 × 512/2), coronal fat-suppressed sequence T2-weighted imaging (Cor × T2+, TR:4600.00 ms/TE:110 ms/5 mm/Fov 12 cm/256 × 256/2), axial enhanced T1-weighted imaging (A × T1+, TR:500.00 ms/TE:12 ms/5 mm/Fov 12 cm/256 × 256/2), and coronal-enhanced T1-weighted imaging (Cor × T1+, TR:331.00 ms/TE:17.9 ms/5 mm/Fov 12 cm/256 × 256/2) data were analyzed by visual identification and encircling of areas of abnormal signal intensity for each MR section using side-to-side comparison on the screen.

### Histochemical staining

At 12 days after carcinoma cell inoculation and demineralization in EDTA (10%) for 2-3 weeks, the tibiae were embedded in paraffin and 5-μm thick sections were cut using a microtome. The sections were stained with Harris' hematoxylin and eosin (HE) to verify cancer cell infiltration and bone destruction (n = 4/group).

### Drugs

Intrathecal injection (i.t.) of all drugs was accomplished *via *lumbar puncture at level L4-5 under brief halothane anesthesia (n = 10/group).

Fluorocitrate (FC, 1 nmol in 10 μl; Sigma), a reversible glial metabolic inhibitor, inhibits aconitase, a Kreb's cycle enzyme expressed in glia, but not neurons [27]. FC was initially dissolved in 2 M HCl and then diluted in 10 mM phosphate-buffered saline (PBS, pH 6.0). At 9 days after carcinoma cell inoculation, rats received an intrathecal (i.t.) injection of FC (1 nmol, group FC) or vehicle (0.3% 2 M HCl in PBS, pH 6.0, group FC vehicle sham). Hind paw withdrawal threshold (PWT) for mechanical stimulation was measured using a von Frey filament at 1 h before FC administration (baseline), and at 1, 2, 4, 6, 8, 10, 12, and 24 h after FC administration, as well as 3, 6, 9, 12, 15, and 18 d after carcinoma cell inoculation.

U0126 (a MEK inhibitor) was dissolved in 5% DMSO and injected i.t. at 0.1 μg/10 μl (group U0126-0.1), 1.0 μg/10 μl (group U0126-1.0), and 10 μg/10 μl (group U0126-10) on day 9 after carcinoma cell inoculation. PWT was measured 1 h prior to U0126 administration (baseline) and at 1, 3, 6, 9, 12, and 24 h after U0126 administration and at 3, 6, 9, 12, 15, and 18 d after carcinoma cell inoculation.

The FC and U0126 doses were based on preliminary experiments (data not shown) and on previous studies of inflammatory pain [28,29]. FC vehicle (0.3% 2 M HCl in PBS, pH 6.0, group FC vehicle sham) or U0126 vehicle (5% DMSO, group U0126 vehicle sham) was used as a positive control, and 10 μl was injected i.t. at the same times as FC or U0126 administration, as described above. For the negative controls, 10 μl of FC (1 nmol in 10 μl, group FC model sham), or U0126 (10 μg/10 μl, group U0126 model sham) were injected i.t. at the same time as FC or U0126, as described above, following inoculation of 10 μl Hank's solution.

### Mechanical allodynia through the use of von Frey filaments

The 50% paw withdrawal threshold (PWT) to a static mechanical stimulus was assessed using von Frey filaments and the up-and-down method [30] which just like we described previously [10,11]. The PWT was measured pre-surgery (baseline, BL) and on days 3, 6, 9, 12, 15, and 18 after cell inoculation. The behavioral test investigator was blinded to group assignments.

### Immunohistochemistry

At 2 h after behavioral testing, the rats (n = 5/group) from the N2 group (normal control rats), K2 group (heat-killed carcinoma cells in 10 μl Hank's solution), A2(d3) group (sacrificed 3 days after inoculation of *in vitro *Walker 256 cells), as well as A2(d6) group, A2(d9) group, A2(d12) group, and A2(d18) group (sacrificed at day 6,9,12 and 18 respectively) were deeply anesthetized with sodium pentobarbital (60 mg/kg i.p.). The histochemistry of double immunofluorescence was performed 10-μm thick, free-floating, L4-5 spinal cord sections as we previously described [28]. Mouse anti-neuronal nuclei (NeuN) Alexa Fluor 488-conjugated monoclonal antibody (neuronal marker, 1:100, Chemicon/Millipore, USA.) was used to label the neurons. Mouse anti-rat OX-42 FITC-conjugated monoclonal antibody (1:10; Chemicon/Millipore, USA) was used to label CR3/CD11b on activated microglia. Mouse anti-glial fibrillary acidic protein (GFAP) Alexa Fluor 488-conjugated monoclonal antibody (1:1000; Chemicon/Millipore, USA) was used to label astrocytes. Anti-phosphorylated ERK (p-ERK, 1:2000; Santa Cruz, USA) was used to detect ERK activation. For double immunolabeling of p-ERK/NeuN, p-ERK/OX-42, and p-ERK/GFAP, sections were incubated in a mixture of antibodies. The sections were rinsed and incubated in Alexa Fluor 594-conjugated secondary antibodies for 3 h (Molecular Probes, 1:200, USA). Digital images were captured from 5 randomly selected sections per animal, and 3 squares (250 × 250 μm) per section were chosen. The stained sections were analyzed using laser-scanning confocal microscopy (Leica, Germany) and fluorescent microscopy (Olympus CKX61, Japan). In addition, for the quantification, the numbers (NUM) of GFAP, OX-42, and p-ERK immunofluorescence-stained single-or double-labeled positive cells in each image was counted with a computer-assisted image analyzer (Image Pro-plus 6.0, Kodak, USA). All measurements were performed by an author who was blind with respect to treatments. The results were averaged for each individual rat. To test immunohistochemistry specificity, primary or secondary antibodies were omitted. Under these conditions, staining corresponding to the second primary antibody was not observed.

### Western blot analysis

Animals (n = 5/time group) were sacrificed rapidly in a CO_2 _chamber. The L4-5 dorsal horns were quickly removed by hydraulic extrusion with saline. The lumbar cord was immediately frozen and maintained at -80°C [9]. Western blot analysis of the p-ERK and p-CREB was performed as previously described [11,12]. Among this, quivalent amounts of protein (30 μg) were used and a rat monoclonal antibody against total ERK or CREB antibody (1:1000; Santa Cruz) was used as the loading control. Western blot analysis was performed as previously described [31]. Scanning densitometry was used for semi-quantitative analysis with a computer-assisted image analyzer (Gel Pro Analyzer, USA).

The p-ERK and p-CREB were measured on day 3, 6, 9, 12, and 18 following cell inoculation (n = 5/time group). In addition, on day 9, rats were sacrificed 6 h after U0126 (10 μg) or vehicle (5% DMSO) intrathecal injection, or at 8 h after FC or vehicle (0.3% 2 M HCl/PBS) injection. The effective dose of U0126 (10 μg) and time point (6 and 8 h each) was based on pain-related behavioral experiments (see results from anti-nociceptive effect of intrathecal U0126 and FC). For the control, U0126/FC vehicle sham, and U0126/FC model sham groups (n = 4/group) were sacrificed, and p-ERK and p-CREB were measured at 6 h or 8 h, respectively.

### Statistical analyses

Statistical analysis was performed using software SPSS 16.0 (SPSS Inc). All data are expressed as mean ± standard error of mean (SEM). Data from the immunohistochemical analysis and Western blots studies were accomplished using a one-way analysis of variance (ANOVA) followed by *post hoc *Dunnett testing. Data from the nociceptive tests were analyzed using ANOVA followed by *Bonferroni *testing for tactile allodynia testing. A value of *P *< 0.05 was considered significant. The individual investigators responsible for behavioral testing and immunohistochemical quantification were blinded to the experimental situation.

## List of abbreviations

CIBP: cancer-induced bone pain; ERK: extracellular signal-regulated protein kinase; CREB: cAMP response element-binding protein; CNS: central nervous system; MAPK: mitogen-activated protein kinase; DRG: dorsal root ganglia; SNL: Spinal nerve ligation; SPECT: single photo emission-computed tomography; MRI: Magnetic Resonance Imaging; HE: Harris' hematoxylin and eosin; FC: Intrathecal injection, i.t; Fluorocitrate; PWT: paw withdrawal threshold; GFAP: glial fibrillary acidic protein; CCI: chronic constriction injury; BDNF: brain-derived neurotrophic factor;interleukin-1β, IL-1β; TNF-α: tumor necrosis factor-α; TLR4: Toll-like receptor 4.

## Competing interests

The authors declare that they have no competing interests.

## Authors' contributions

Li-na Wang, Role:study design, data colletion and analysis, and manuscript preparation.

Ming Yao, Role: Carry out the animal surgery, behavior testing and radiological bone examination.

Jian-ping Yang, Role: conduct of the study, and participate in its design and coordination.

Jun Peng, Role: data analysis.

Yan Peng, Cai-fang Li and Yan-bing Zhang, Role: Carry out the animal surgery and behavior testing.

Hao Cheng and Fu-hai Ji, Role: Carried out western blot analysis.

Qi-nian Xu, Xiu-yun Wang and Jian-ling Zuo, Role: Carry out the Walker 256 mammary gland carcinoma cell line preparation.

All authors have read and approved the final manuscript.

## Supplementary Material

Additional file 1 **paw withdrawal latency (PWL) test following a thermal nociceptive stimulus**. Before Walker 256 cell injection, the overall mean baseline paw withdrawal latency (PWL) to noxious heat stimuli (using a previously described method [Hargreaves, et al. Pain 32, 77-88.] [Lina W, et al. Brain Res 2006; 1120:46-53.]) was similar in all groups of rats and there was no significant difference in PWL between left and right hind paws. Following injection of Walker 256 cells cultured *in vitro *(group V1) and *in vivo *(group A1) into the left tibia, the left hind paw PWL significantly and progressively decreased between days 3 and 6 compared with that of the contralateral hind paw and of the hind paws of groups N1 and K1, which remained at the pre-injection level (* *P *< 0.05 ** *P *< 0.05 *vs *Group N1; # *P *< 0.05 *vs *Group K1, see Additional file 1). However, *post-hoc *means comparisons revealed that Walker 256 cell inoculation of the tibia induced no significant (*P *> 0.05) decrease of PWL on days 9, 12, 15 and 18 after inoculation compared with groups N1 and K1. Click here for file
