# Supplementary figures and images for: Cancer-induced bone pain sequentially activates the ERK/MAPK pathway in different cell types in the rat spinal cord
Source: Mol Pain. 2011 Jul 1;7:48. doi: 10.1186/1744-8069-7-48 (PMC3150304; doi:10.1186/1744-8069-7-48)

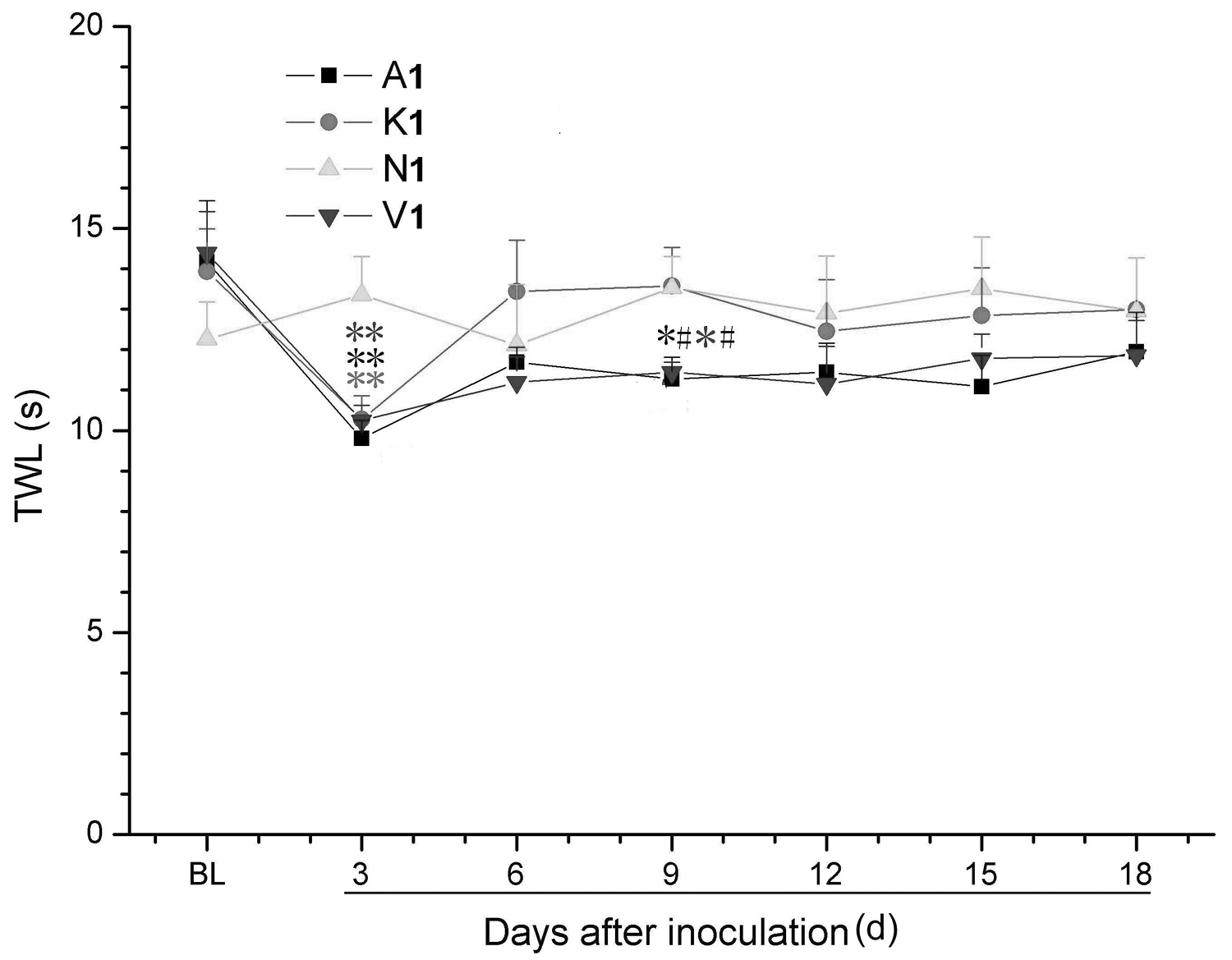

Supplement: Additional file 1 — paw withdrawal latency (PWL) test following a thermal nociceptive stimulus. Before Walker 256 cell injection, the overall mean baseline paw withdrawal latency (PWL) to noxious heat stimuli (using a previously described method [Hargreaves, et al. Pain 32, 77-88.] [Lina W, et al. Brain Res 2006; 1120:46-53.]) was similar in all groups of rats and there was no significant difference in PWL between left and right hind paws. Following injection of Walker 256 cells cultured in vitro (group V1) and in vivo (group A1) into the left tibia, the left hind paw PWL significantly and progressively decreased between days 3 and 6 compared with that of the contralateral hind paw and of the hind paws of groups N1 and K1, which remained at the pre-injection level (* P < 0.05 ** P < 0.05 vs Group N1; # P < 0.05 vs Group K1, see Additional file 1). However, post-hoc means comparisons revealed that Walker 256 cell inoculation of the tibia induced no significant (P > 0.05) decrease of PWL on days 9, 12, 15 and 18 after inoculation compared with groups N1 and K1. [file 1744-8069-7-48-S1.TIFF]
